# Emotional decision-making in autism spectrum disorder: the roles of interoception and alexithymia

**DOI:** 10.1186/s13229-016-0104-x

**Published:** 2016-10-13

**Authors:** Punit Shah, Caroline Catmur, Geoffrey Bird

**Affiliations:** 1 Department of Neuroimaging, Institute of Psychiatry, Psychology and Neuroscience, King’s College London, University of London, London, SE5 8AF UK; 2 Department of Psychology, Institute of Psychiatry, Psychology and Neuroscience, King’s College London, University of London, London, UK; 3 Institute of Cognitive Neuroscience, University College London, London, UK; 4 MRC SGDP Centre, Institute of Psychiatry, Psychology and Neuroscience, King’s College London, University of London, London, UK

**Keywords:** Autism, Emotion, Replication, Insula, Alexithymia, Interoception, Framing effect, Decision-making

## Abstract

The way choices are framed influences decision-making. These “framing effects” emerge through the integration of emotional responses into decision-making under uncertainty. It was previously reported that susceptibility to the framing effect was reduced in individuals with autism spectrum disorder (ASD) due to a reduced tendency to incorporate emotional information into the decision-making process. However, recent research indicates that, where observed, emotional processing impairments in ASD may be due to co-occurring alexithymia. Alexithymia is thought to arise due to impaired interoception (the ability to perceive the internal state of one’s body), raising the possibility that emotional signals are not perceived and thus not integrated into decision-making in those with alexithymia and that therefore reduced framing effects in ASD are a product of co-occurring alexithymia rather than ASD per se. Accordingly, the present study compared framing effects in autistic individuals with neurotypical controls matched for alexithymia. Results showed a marked deviation between groups. The framing effect was, in line with previous data, significantly smaller in autistic individuals, and there was no relationship between alexithymia or interoception and decision-making in the ASD group. In the neurotypical group, however, the size of the framing effect was associated with alexithymia and interoception, even after controlling for autistic traits. These results demonstrate that although framing effects are associated with interoception and alexithymia in the neurotypical population, emotional and interoceptive signals have less impact upon the decision-making process in ASD.

The way options are framed can induce bias in decision-making. Options presented in a “gain” frame (you keep $30 of an initial $50) are preferred to mathematically equivalent options presented in a “loss” frame (you lose $20 of an initial $50). The increase in participants choosing the option when in the gain frame compared to the loss frame is known as the “framing effect” (FE [1]). FEs index the influence of emotion on decision-making, with converging evidence for the role of prefrontal-amygdala circuity [2, 3] and insular cortex [4].

Studying FEs in clinical groups helps explicate its neurocognitive basis [5] and may aid the design of interventions to reduce symptoms related to abnormal decision-making. FEs have therefore been investigated in anxiety [6] and in one previous study [7] in autism spectrum disorder (ASD). The present study re-examines FEs in individuals with ASD after accounting for impaired interoception (perception of the state of one’s own body). This is necessary due to recent claims of atypical interoception in ASD [8–12] and of a role for interoception in decision-making [13, 14]. It was previously found that autistic^1^ individuals are less susceptible to FEs because they do not incorporate emotional information into decision-making. It was suggested that, although this may enhance logical consistency, insensitivity to emotional signals during decision-making may also underpin putative core deficits in ASD (such as impaired empathy and emotion recognition).

The characterization of ASD as primarily an affective disorder has been questioned due to recent research highlighting the co-occurrence of autism and alexithymia. Alexithymia is characterized by difficulties identifying and describing one’s own emotions, and recent work suggests that it is best characterized as a general interoceptive impairment [15–19]. Elevated rates of alexithymia are observed in ASD [20–22] and other clinical conditions, including anxiety, depression, and eating disorders [23–25]. Importantly, however, 10 % of the population are estimated to have alexithymia in the absence of any psychiatric or neurological condition. Alexithymia is therefore an independent construct with its own genetic and neurocognitive basis [26, 27], instead of being a feature or symptom of other clinical disorders. This means that groups with and without ASD can be matched for alexithymia (e.g., using the 20-item Toronto Alexithymia Scale [TAS-20] [28]) to examine the relative contributions of ASD and alexithymia to socio-emotional functioning. Using this methodology, several studies now demonstrate that co-occurring alexithymia is responsible for many of the affective impairments previously thought to be due to ASD [29–33]. The finding that alexithymia, and not ASD per se, is responsible for affective impairments raises the question of whether the reduction in FEs previously attributed to ASD is in fact due to alexithymia. The question is especially pertinent when one considers alexithymia to be a general impairment of interoception because interoceptive ability, driven by insula activity [34], has been demonstrated to moderate the effects of emotion on decision-making (e.g., [13]). This suggestion is consistent with theories postulating that the insula integrates intero-and exteroceptive signals about uncertainty to guide decision-making [35, 36].

More recently, however, research has shown divergent roles of autism and alexithymia across high-level judgment and decision-making tasks that invoke emotional processing. For example, Brewer and colleagues [37] examined moral decision-making in those with and without ASD as a function of alexithymia. Existing models of moral reasoning posit two routes by which one can arrive at a judgment of moral acceptability—one based on emotional information driven by empathy for the victim’s distress and the other based on societal rules relating to the acceptability of specific behaviors. In those without ASD, moral judgments were based on emotional information, and increasing levels of alexithymia led to increasingly atypical judgments. In those with ASD, however, emotional information was not used to make moral judgments, and therefore moral judgments were not impacted by levels of alexithymia. This finding suggests that alexithymia may have a different effect on judgment and decision-making in people with and without ASD.

It is clear that there is theoretical impetus to investigate emotional decision-making in ASD while accounting for the contribution of alexithymia and interoception. The present study therefore compared the magnitude of the FE between individuals with ASD and an alexithymia-matched control group. We hypothesized that there would be a difference in the FE between individuals with and without ASD (see [7]) and also that alexithymia and interoception would be associated with the magnitude of the FE. However, following recent evidence [37–39], it was also predicted that the relationship between interoception, alexithymia, and the FE may be reduced in autistic individuals relative to neurotypical individuals.

## Method

### Participants

A power analysis [40] was conducted to inform sample size considerations based on the large group difference in the FE (Cohen’s *d* = 3.62) observed by De Martino et al. [7]. This indicated that four participants were required per group where power = 0.95, *α* = 0.05. However, in order to ensure there was adequate variance on autism and alexithymia scores, and perform regression analyses controlling for confounding variables, 42 individuals with (*n* = 21) and without (*n* = 21) ASD participated in the study. This sample size also served to ensure the ASD and control groups could be matched on demographic variables and alexithymia (see below). Two participants were excluded. In the control group, one participant was excluded because their framing effect was >3 SD above the group mean. In the ASD group, one participant failed to respond on >50 % of trials and so did not complete the task. Therefore, a total of 40 participants were included in the final analysis. Twenty participants with ASD were age, IQ [41–43], and gender matched with 20 neurotypical controls (Table 1).

**Table 1:** Participants and group matching

	ASD	Controls	Comparison
*N*	20	20	–
Gender	17 males, 3 females	14 males, 6 females	*χ* ^2^(1) = 1.29, *p* = 0.26
Mean age (years)	32.70 (11.18)	34.10 (14.20)	*p* = 0.74
Mean full-scale IQ	108.55 (12.80)	110.95 (13.62)	*p* = 0.57
Mean AQ	35.45 (7.47)	20.80 (8.94)	*p* < 0.001
Mean TAS-20	58.11 (13.00)	55.37 (18.33)	*p* = 0.63
Number of participants with/without alexithymia	8/12	11/9	*χ*2(1) = 0.90, *p* = 0.34
Mean TA	53.75 (10.15)	47.80 (14.09)	*p* = 0.13
Mean SA	39.90 (9.62)	36.80 (10.47)	*p* = 0.34
ADOS classification	9 autism, 11 autism spectrum	–	–
Mean ADOS-G score	9.50 (2.44)	–	–

*Note*. Mean age, gender, autism-spectrum quotient (AQ), 20-item Toronto Alexithymia Scale (TAS-20), (state (SA) and trait (TA)) anxiety scores, and IQ scores for the autism spectrum disorder (ASD) group and the matched neurotypical controls. Autism Diagnostic Observational Schedule (ADOS) score and classification details for the ASD group: A higher score represents greater autism severity. Standard deviations are shown in brackets. The IQ score for one control participant was unavailable and therefore not reflected in the group mean

All autistic participants were recruited from a database of volunteers that have received a clinical diagnosis of ASD from an independent clinician (as per the Diagnostic and Statistical Manual of Mental Disorders, 4th Edition [44]). Diagnosis was confirmed using module 4 of the Autism Diagnostic Observational Schedule (ADOS [45]) by a research-reliable administrator, and all participants in the ASD group met criteria for autism or autism spectrum disorder. Control participants were recruited from a database of volunteers who reported no known psychiatric, neurological, or neurodevelopmental disorder (including ASD). All participants completed the Autism-spectrum Quotient (AQ [46]) as a measure of autistic traits. The ASD group scored significantly higher on this measure than the control group, in line with previous literature [46].

Importantly, participants also completed the 20-item Toronto Alexithymia Scale (TAS-20 [28]). Each group contained individuals with (TAS-20 score of ≥61), and without, alexithymia, such that the ASD and control groups were matched on trait alexithymia (see Table 1). Participants had normal or corrected-to-normal vision and gave informed consent prior to participation. Ethical clearance was granted by the local ethics committee.

## Materials and procedure

### Emotional decision-making task

**Fig. 1:**
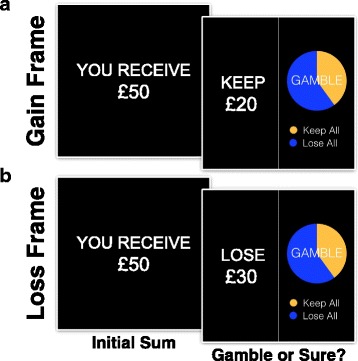
Measuring the framing effect. Participants are shown the sum of money received to play in that trial for 2 s (e.g., “you receive £50”). They are required to choose between a “gamble” or “sure” option within 4 s. The “sure” option is presented as either **a** the sum of money retained from the initial starting amount (e.g., keep £20 of £50—gain frame) or **b** money lost from the initial amount (e.g., lose £30 of £50—loss frame). The “gamble” is identical in both frames and represented by a pie-chart showing the probability of winning (*yellow*) or losing (*blue*). Feedback about trial outcomes is not presented during the experiment

The FE was measured using the financial decision-making task first reported in 2006 [2] and used by De Martino et al. in 2008 [7]. It is widely used to study emotional decision-making and has shown to be a robust and replicable measure of the FE [47]. At the beginning of each trial, participants are shown a sum of money (e.g., “you receive £50”) for 2 s. Four different starting amounts are used: £100, £75, £50, and £25. Participants are informed that they are unable to keep the total sum of money and given 4 s to decide between a “sure” and a “gamble” (risky) option using a response keypad. If the participant did not respond within 4 s, the trial was omitted from analysis. Crucially, the sure option is presented in either a “gain” frame as the sum of money retained from the initial starting amount (e.g., keep £20 of £50) or “loss” frame as money lost from the initial amount (e.g., lose £30 of £50). The “gamble” is identical in both frames and represented by a pie-chart showing the probability of winning or losing (Fig. 1). Four different probabilities were used in the experiment: 20, 40, 60, and 80 %. The expected value of the options was balanced in each trial (except for catch trials, see below) and mathematically equivalent between the sure option and the gamble. For example, if participants initially received £50, they were then required to choose between the options “keep £20” or a gamble with a 40 % chance of winning £50 and a 60 % chance of winning nothing. The response to the framing manipulation was first measured for each individual in each group, by calculating the percentage of trials in which participants chose the “gamble” versus “sure” option within each frame. The size of each individual’s FE was computed by taking the difference between the percentage of trials in which they gambled in the gain from the loss frame; therefore, greater susceptibility to FEs is denoted by a larger value.

Given the equivalence of the choices in terms of expected outcomes, “catch” trials (32 in each session) were included to ensure that participants remained engaged in the task. In these catch trials, within both frames, expected outcomes for the sure and gamble option were clearly unbalanced: in half of the trials (“gamble weighted”), the gamble option was preferable (e.g., 95 % probability of winning by taking the gamble option vs a sure choice of 50 % of the initial amount), and for the other half of trials (“sure weighted”), the sure option was preferable (e.g., 5 % probability of winning by taking the gamble option vs a sure choice of 50 % of the initial amount). As in the main experimental trials, the catch trials were also presented in either a gain or a loss frame.

### Measuring interoception and time estimation

Interoceptive accuracy (IA; see [48, 49]) was measured using the Heartbeat Tracking task [50]. Participants were seated, required to close their eyes, and silently count their heartbeats during four intervals (25, 35, 45, and 100 s). The order of intervals was randomized across participants. Participants were instructed not to measure their pulse by any means other than “concentrating on their heart beats.” Heartbeat signals were acquired using a pulse oximeter (Contec Systems CMS50D+; Qinhuangdao, China) attached to the left index finger, while the right arm was placed on the table.

Performance on the Heartbeat Tracking task may be influenced by one’s ability to sustain attention during counting tasks and/or accurately estimate time [51]. Participants were therefore instructed to judge the duration of three randomized intervals (19, 37, and 49 s; e.g., [52]). Performance on the Time Estimation (TE) task is known to follow a similar distribution to the Heart Beat Tracking task in participants with and without ASD, thereby indicating the tasks are matched in difficulty [16]. The TE task was performed by participants while remaining in the position used for the interoception task.

IA was quantified on a scale between 0 and 100 % using a standard transformation: 1/4 Σ(1-(|recorded number of heartbeatsinterval − counted number of heartbeats_interval_|/recorded heart beats_interval_)) × 100. Higher scores are indicative of better IA. TE score was also computed on a scale of 0 to 100 % using the following formula: 1/3 Σ(1-(|estimated elapsed time_﻿interval﻿_ − actual elapsed time_interval_|/actual elapsed time_interval_)) × 100. Higher scores are indicative of better TE ability.

### Confounding variables

A number of clinical conditions have been associated with interoception (see [53]). Anxiety has been most closely associated with IA [54] and the FE [55]; therefore, in addition to the TAS-20 and AQ, participants completed the Spielberger State/Trait Anxiety Inventory ([56]; Table 1).

### Procedure

The study was conducted in a dimly lit and quiet testing cubicle. Participants completed the questionnaires immediately before the Heart Beat Tracking/TE tasks. The order in which the interoception and TE tasks were completed was counterbalanced across participants. After familiarization with the decision-making task and several practice trials, the participants began the decision-making procedure. This was divided into three sessions of 96 pseudorandomized trials, each comprising 32 loss frames, 32 gain frames, and 32 catch trials. The percentage of the money offered, total starting amount, and number of trials per session were counterbalanced between frame conditions. Each session was 17 min in duration and interleaved with a compulsory rest period (3 min). The complete procedure was approximately 1 h long and presented using *PsychoPy* [57, 58] on a 22-in. Samsung SyncMaster 2233RZ LCD monitor (resolution of 1680 × 1050 and refresh rate of 120 Hz).

### Data analysis

Data were analyzed in three ways. First, group-wise analyses compared individuals with and without an ASD diagnosis. Second, correlational analyses were used to determine the relationships between interoception, alexithymia, autism, and the FE. Finally, moderation analyses examined whether the relationships between interoception, alexithymia, and the FE were different in the ASD and control groups.

## Results

We initially established that, in total, 0.31 and 0.80 % of trials were excluded for control and autistic participants, respectively. There was no significant group difference in the number of excluded trials, *t*(38) = 1.43, *p* = 0.17, *d* = 0.45, 95 % CI for *d*[−0.17, 1.08].^2^ There was also no significant group difference between the ASD and control groups in IA (control: *M* = 70.99, *SD* = 20.40, ASD: *M* = 65.54, *SD* = 25.85, *t*(38) = 0.74, *p* = 0.46, *d* = 0.23, 95 % CI for *d*[−0.39, 0.85]). However, autistic participants significantly outperformed the control group on the TE procedure (control: *M* = 72.40, *SD* = 19.96, ASD: *M* = 85.24, *SD* = 11.55, *t*(38) = 2.49, *p* = 0.017, *d* = 0.79, 95 % CI for *d*[−0.14, 1.43]).

**Fig. 2:**
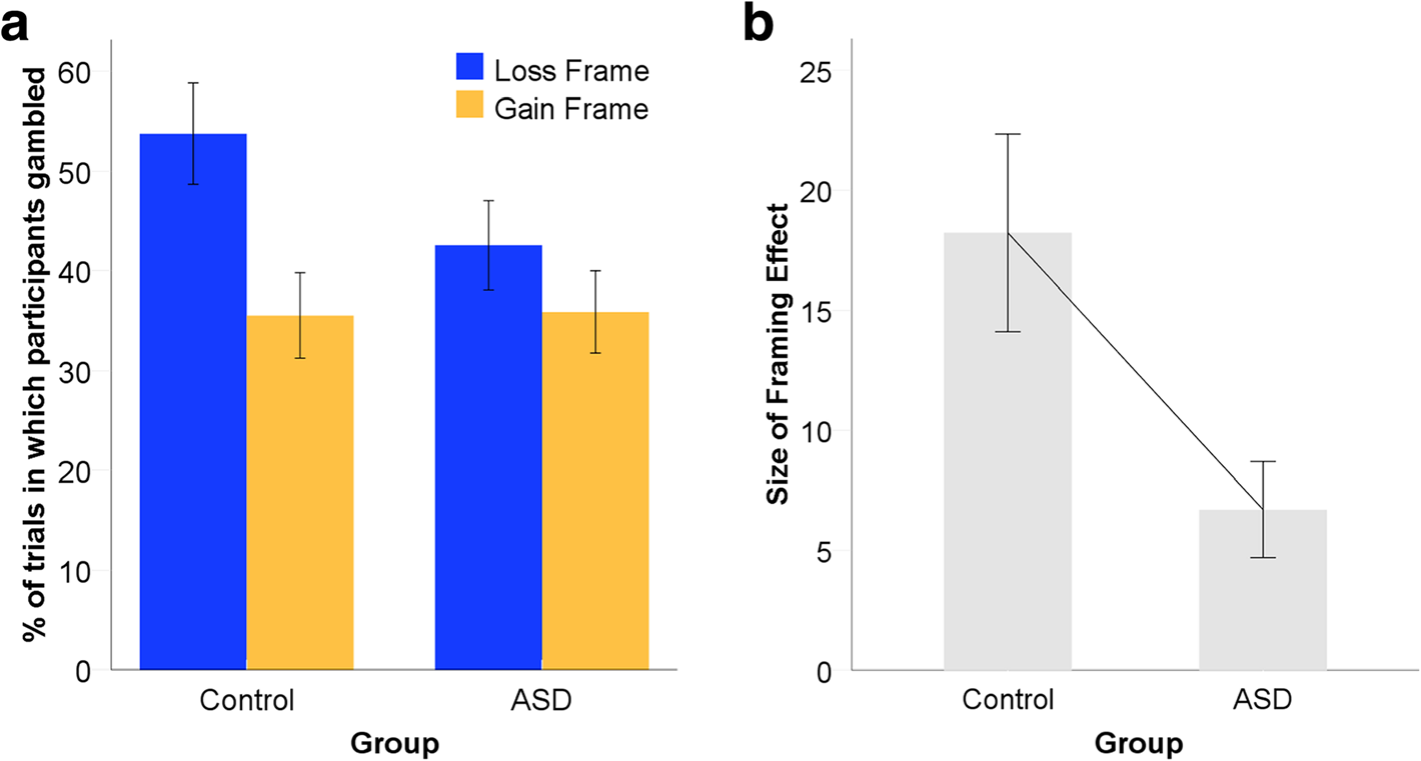
The framing effect (FE) by group. **a** The percentage of trials in which participants chose to gamble in the Loss compared with the Gain frame. Although both controls and autistic participants gambled significantly more in the loss than gain frame, the size of **b** the FE (i.e., the difference between gambling in the loss and gain frame) was significantly larger in the control group. *Error bars* denote ±1 SEM

FEs were evident in both individuals with and without ASD, as demonstrated by the greater percentage of trials in which participants chose the gamble option in the loss than gain frame (Fig. 2a). However, the size of the FE (i.e., the difference between gambling in the Loss compared with Gain Frame) was substantially smaller and less variable in the ASD compared with the control group (Fig. 2b).

These data were analyzed using mixed model ANOVA with frame (gain, loss) as the within-subjects factor and group (ASD, control) as the between-subjects factor. There was no main effect of group, *F*(1, 38) = 0.83, *p* = 0.37, η_p_^2^ = 0.02 [90 % CI = 0, 0.05], indicating there was no overall group difference in the number of trials on which they chose to gamble. There was a large and significant effect of frame, *F*(1, 38) = 29.59, *p* < 0.001, η_p_^2^ = 0.44 [90 % CI = 0.23, 0.57], which confirmed the effectiveness of the framing manipulation. Crucially, the group × frame interaction reached significance, *F*(1, 38) = 6.35, *p* = 0.016, η_p_^2^ = 0.14 [90 % CI = 0.02, 0.31], which indicated that susceptibility to the framing manipulation was different between those with and without ASD. Planned comparisons confirmed both control *t*(19) = 4.42, *p* < 0.001, *d* = 1.43, 95 % CI for *d*[0.71, 2.14], and autistic *t*(19) = 3.34, *p* = 0.003, *d* = 1.08, 95 % CI for *d*[0.39, 1.76], participants gambled significantly more in the loss compared with the gain frame. Importantly, however, the size of the FE was significantly larger in the control group than in the ASD group, *t*(38) = 2.52 *p* = 0.016, *d* = 0.80, 95 % CI for *d*[0.15, 1.44]. This result supported our first hypothesis.

Data from catch trials were inspected to examine whether the participants were engaged in the task and to assess if the ASD participants were responding to monetary incentives in a manner that was comparable to the control group. The analysis of catch trials also determined whether overall risk tendency was matched across groups. Only one participant in each group showed an atypical pattern of responding, indicative of extreme aversion to risk (i.e., choosing not to gamble on trials when there was a 95 % probability of keeping the initial amount compared to 50 % probability of a win if they chose the “sure” choice). However, the pattern of significance detailed above remained when both individuals were excluded from analysis (e.g., group × frame interaction; *F*(1, 36) = 6.38, *p* = 0.016, η_p_^2^ = 0.15 [90 % CI = 0.02, 0.32]). Furthermore, re-including all participants, and in line with previous data [7], the number of catch trials on which participants gambled was not different between groups, *t*(38) = 0.94, *p* = 0.35, *d* = 0.30, 95 % CI for *d*[−0.33,0.92]. The critical group × frame interaction also remained significant after including individual performance on catch trial data as a covariate in the analysis, *F*(1, 37) = 5.40, *p* = 0.026, η_p_^2^ = 0.13 [90 % CI = 0.01, 0.29].

In line with the existing literature (e.g., [16]), there was an association between IA and the TAS-20, across groups (*r* = −0.58, *p* < 0.001), in the control group (*r* = −0.60, *p* = 0.005), and ASD group (*r* = −0.61, *p* = 0.004). Supporting our second hypothesis, after collapsing across groups, the size of the FE was negatively correlated with both autistic traits (*r* = −0.43, *p* = 0.005) and alexithymia scores (*r* = −0.36, *p* = 0.023). Conversely, there was a positive correlation between IA and the FEs (*r* = 0.37, *p* = 0.019) but no such relationship with TE ability (*r* = 0.02, *p* = 0.92).

We conducted moderation analyses to investigate whether the relationship between interoception and the FE and the association between alexithymia and the FE (i) significantly varied as a function of group and (ii) held after controlling for potentially confounding variables. Participant age, IQ, state, and trait anxiety scores (see Table 1), TE ability, group (ASD, control), and alexithymia (TAS-20 scores) were entered as predictor variables into step 1 of a hierarchical regression. Group was the only significant predictor of the FE (*β* = 0.43, *t* = 2.43, *p* = 0.021), whereas alexithymia (*β* = −0.27, *t* = −1.68, *p* = 0.10) and the other variables failed to reach significance (other *p*s > 0.16). Together, step 1 explained 32.90 % of variance in the FE. When the alexithymia × group term was added to the model in step 2, the results differed such that in addition to group (*β* = 0.41, *t* = 2.49, *p* = 0.019), both state (*β* = −0.52, *t* = −2.29, *p* = 0.029) and trait (*β* = 0.48, *t* = 2.05, *p* = 0.049) anxiety scores were significant predictors of a larger FE. Again, alexithymia and other confounding variables remained non-significant (*p*s > 0.34). Most importantly, however, the predictor coding the interaction between alexithymia and group was a significant predictor of the FE (*β* = −0.40, *t* = −2.27), significantly increasing the variance accounted for by 9.80 %, *F*(1, 30) = 5.14, *p* = 0.031. This analysis confirmed that the relationship between alexithymia and the FE was moderated by group.

Alexithymia and IA were highly correlated. In order to avoid problems with multicollinearity, a second moderation analysis was conducted with IA replacing alexithymia in steps 1 and 2, and accordingly, an IA × group interaction term in step 2. Group was a significant predictor of the FE (*β* = 0.41, *t* = 2.43, *p* = 0.021), as was IA (*β* = 0.33, *t* = 2.13, *p* = 0.041). The other variables in the first step failed to reach significance (other *p*s > 0.23) and altogether, step 1 explained 36.1 % of variance in the FE. In step 2, both group (*β* = 0.41, *t* = 2.57, *p* = 0.015) and IA (*β* = 0.40, *t* = 2.68, *p* = 0.012) remained as significant predictors. More importantly, the interaction between these variables was also a predictor of the FE (*β* = 0.32, *t* = 2.27), significantly increasing the variance accounted for by 9.40 %, *F*(1, 31) = 5.17, *p* = 0.030, and thus confirmed that the relationship between interoception and the FE was different between groups. These patterns of results supported our third hypothesis, which also held when both moderation analyses were repeated without including age, IQ, TE ability, and anxiety scores (this analysis which just includes the variables of key interest may be more appropriate given the sample size).

**Fig. 3:**
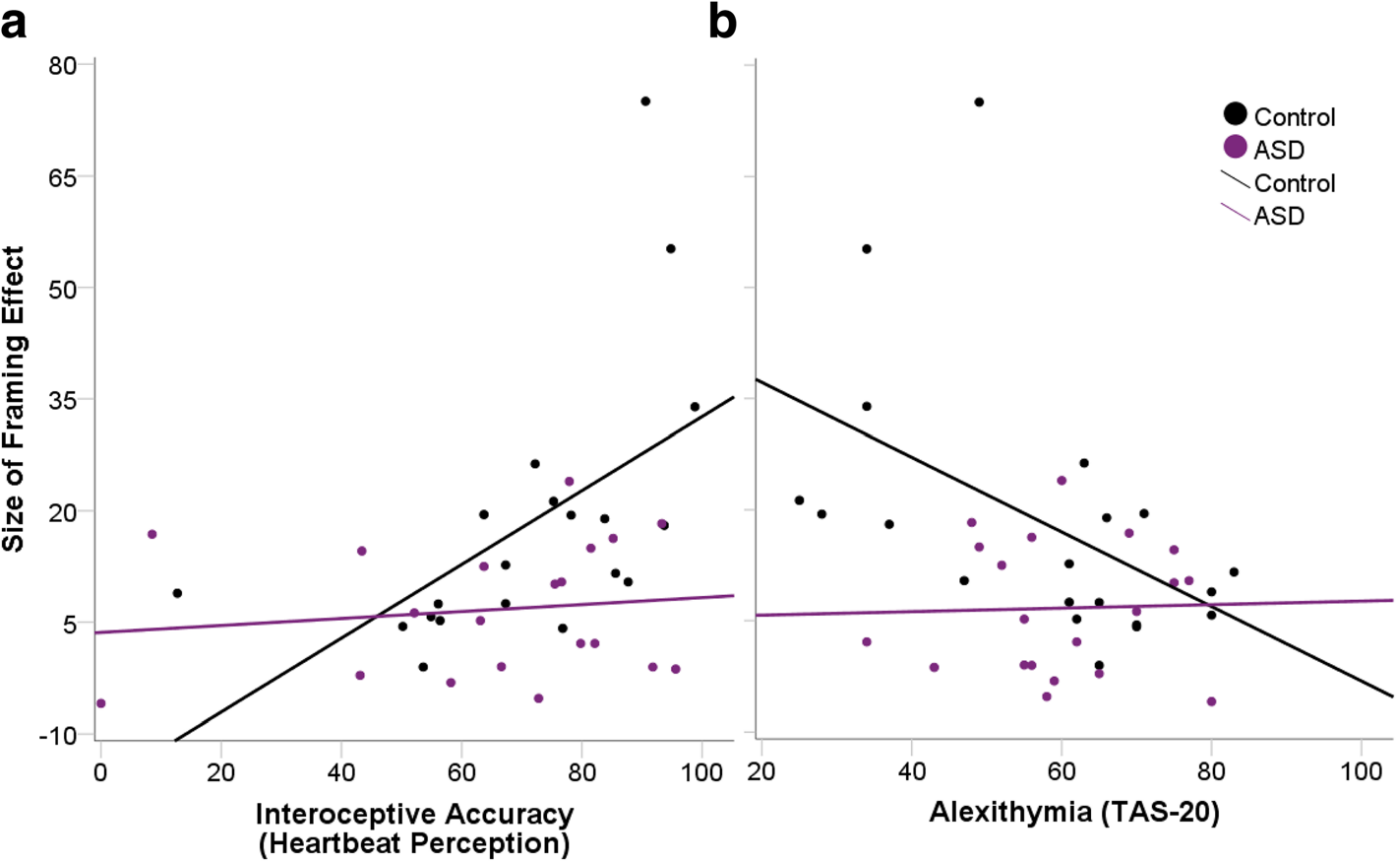
Associations between interoception, alexithymia, and framing effects. **a** Individuals with greater interoceptive accuracy (IA) showed a larger FE as shown by the correlation between IA and the FE in the control group (*r* = 0.55, *p* = 0.012). No such relationship was observed in the ASD group (*r* = 0.14, *p* = 0.57). **b** The correlation between the TAS-20 and the FE was not significant in the ASD group (*r* = 0.03, *p* = 0.89), but was in the control group (*r* = −0.49, *p* = 0.029)

Correlational analyses showed that both the relationship between alexithymia and the FE and the association between interoception and the FE were only observed in, and therefore being driven by, the control group (Fig. 3). Greater IA (*r* = 0.55, *p* = 0.012) and lower alexithymia scores (*r* = −0.49, *p* = 0.029) were associated with a larger FE. The FE was not associated with any of the other demographic or potentially confounding variables (all *p*s > 0.11). In contrast, there was no relationship between alexithymia and the FE (*r* = 0.03, *p* = 0.89), or interoception and the FE (*r* = 0.14, *p* = 0.57) in the ASD group; however, there was a negative association between age and the size of the FE (*r* = −0.69, *p* = 0.001).

## Discussion

The present study investigated whether individuals with ASD exhibit enhanced logical consistency, i.e., smaller FEs, after accounting for alexithymia and interoception. More generally, we sought to investigate the relationship between interoception and decision-making which has seldom been investigated in typical individuals and never before in a clinical group. To these ends, a well-established measure of the FE was used, together with a widely used measure of interoception. Replicating a previous study [7], FEs were significantly smaller in autistic individuals compared to the non-autistic control group. Importantly, these results were found even when the control group was matched for alexithymia, suggesting that the enhanced logical consistency seen in the ASD group was due to ASD itself, rather than co-occurring alexithymia.

Moderation and within-group analyses demonstrated a striking difference between the relative impact of alexithymia and interoceptive ability on the ASD and control groups. For individuals without ASD, IA and alexithymia both significantly predicted the size of the FE, such that better interoception (and lower trait alexithymia) was associated with a larger FE. This result is in accordance with the model forwarded by De Martino and colleagues [2] in which emotional information influences decision-making in neurotypical individuals, and the extent to which one can accurately perceive emotional information/arousal determines the influence it has on decision-making (see also [13]). In contrast, alexithymia and interoception did not predict susceptibility to the FE in those with ASD. This differential pattern of results in people with and without ASD indicates the use of different strategies when making judgments: one strategy involving gist-based, fuzzy, intuitive processes used by non-autistic individuals, and another strategy which recruits rule/verbatim-based analysis which is recruited by individuals with ASD (see [59] for a neurodevelopmental perspective). These results are consistent with the idea that, instead of using interoceptive or emotional information, and regardless of whether they have access to these signals, individuals with ASD use a rule-based strategy which results in a smaller FE at the group level (see [60]). These results, in line with our predictions, are consistent with recent research showing divergent roles of autism and alexithymia across high-level judgment and decision-making tasks that invoke emotional processing [37–39].

De Martino et al. [7] proposed that “although this impairment in processing contextual emotional information protects ASD subjects from the framing bias, leading to more consistent behavior in situations of risk, it may come at a cost of the social, emotional, and behavioral deficits that characterize the condition” (p. 10,749). The present study is consistent with this proposal, insofar as it was clearly shown that autistic individuals with social atypicalities are indeed less susceptible to the FE. However, we found that IA and alexithymia were not correlated with the size of FE in ASD. This indicates that use of rule-based strategies, leading to a smaller FE, is less related to social-emotional atypicalities in ASD and may be more closely related to the rigid and repetitive behavioral difficulties in this condition. The (over) reliance on rule-based strategies might lead to difficulties in day-to-day functioning (e.g., insistence on sameness, potentially leading to difficulties gaining or maintaining employment in certain job roles). Equally, however, the reduced use of interoceptive signals—and thereby enhanced logical consistency in ASD—may confer an advantage in many situations where emotional information would otherwise interfere with the optimal outcome (e.g., gambling, financial investments, and risk assessments). More generally, neurocognitive strengths shown by individuals with ASD are both under researched, and/or commonly interpreted as impairments, yet the findings from the present study highlight that certain cognitive processes in ASD may, depending on the situation, benefit as well as impair day-to-day functioning (see also [61–63]). On a related note, it is to be emphasized that, while the current study replicates and extends De Martino and colleagues’ results [7], both studies contained high functioning adult ASD samples which limits the generalizability of these findings. Future research on emotional decision-making, alexithymia, and interoception in lower functioning adults and children with ASD are required, and such research will more comprehensively elucidate the causes and consequences of atypical decision-making on individuals across the autism spectrum.

It was not possible to perform neuroimaging in the present study; however, the current results also accord with existing neuroscience data on the FE. The insula has been shown to be involved in emotional decision-making, interoception, and alexithymia [32, 34–36, 64, 65], which together, is likely to underpin the pattern of results that we observed in the control group. Similarly, the strength of prefrontal-amygdala connectivity, known to be atypical in ASD [66–68], is thought to be positively correlated with the size of the FE. This supports the idea that emotional information has a smaller influence on decision-making in autistic individuals and may explain the smaller FE observed in the ASD group.

When considered as a whole, extant research suggests that both ASD itself and co-occurring alexithymia can contribute to atypical performance in ASD in a task-dependent manner. When interoceptive or emotion processing is necessary and sufficient for accurate performance (e.g., tasks assessing interoception, emotion perception, or empathy), then any “ASD impairment” is likely due to alexithymia, such that individuals with ASD without co-occurring alexithymia are unimpaired [29–33]. However, for socio-cognitive tasks that typically involve, but are not necessarily reliant upon, emotion processing (moral judgments, decision-making, Theory of Mind), then individuals with ASD may perform the tasks atypically (demonstrating either impairments or superior performance) because they use a non-affective strategy to complete the task. Performance on these latter tasks would therefore be determined by the presence of an ASD diagnosis, rather than co-occurring alexithymia.

In addition to the primary aim of the study, these data provide evidence for intact IA in ASD. This accords with a recent study in which ASD and control groups were matched for alexithymia: In line with Shah and colleagues’ data [16], the present study indicated that alexithymia, not autism, is associated with atypical interoception.^3^ Previous studies assessing interoceptive ability in ASD which do not control for alexithymia have produced inconsistent results [8–12], but a clearer picture is obtained when this important source of variance is accounted for [16, 17, 19, 69] A higher incidence of alexithymia in many psychiatric and neurological disorders supports the inclusion of alexithymia questionnaires when studying interoception in any clinical group, in order to decompose any reduced IA into that accounted for by the condition itself and that accounted for by co-occurring alexithymia [16, 17]. More generally, interoception research is typically conducted in high functioning adults; therefore, there is a need for interoception research including a wider range of individuals to firmly establish the existence of interoceptive impairments (if any) in ASD and other clinical groups.

An enhanced TE ability in ASD relative to the control group was also observed in these data. Time perception was not the focus of the current study, and the TE task was designed as a control rather than a sensitive measure of time perception ability. These factors mean that we do not wish to draw strong conclusions from this result, but the finding of superior time perception in autistic adults accords with reports of enhanced time perception in children with ASD [70]. Similarly, the present study was designed to investigate the relative contributions of autism, alexithymia, and interoception to the FE, while controlling for anxiety. Nevertheless, in line with existing research, increased anxiety was associated with larger FEs [6, 55] after accounting for autism and alexithymia. This warrants further investigation in future research with larger samples comprising sufficient variance to fully address the interrelationship between ASD, anxiety, alexithymia, interoception, and emotional decision-making. Finally, although the negative relationship between age and the size of the FE in ASD was unexpected, it seems likely that increased time living with the cognitive features of ASD—such as a bias towards an analytical rule-based strategy when making judgments—may increase the impact of ASD on reduced susceptibility to FEs. Although not observed in this study, there are reports of increased FEs with age in typical populations, suggesting a developmental approach to FEs in ASD, and other clinical groups may prove fruitful [59].

## Conclusions

In summary, the present study replicates De Martino et al.’s finding [7] of enhanced logical consistency in ASD. It was shown that this is unlikely to be due to co-occurring alexithymia—instead evidence suggested that interoceptive ability and alexithymia were unrelated to the FE in individuals with ASD. In contrast, both alexithymia and interoception were associated with the size of FEs in neurotypical individuals, in line with contemporary suggestions that interoceptive and emotional signals guide decision-making under uncertainty [35, 36]. Together, these results reinforce the idea that atypical decision-making is a robust feature of ASD, while highlighting the importance of alexithymia when studying interoception and emotional decision-making in clinical and non-clinical samples.

## Abbreviations

AQ: Autism-spectrum quotient
ASD: Autism spectrum disorder
FE: Framing effect
IA: Interoceptive accuracy
TAS-20: 20-item Toronto Alexithymia Scale
TE: Time estimation

## Footnotes

1 We use the word autistic to refer to individuals with ASD as this terminology is preferred by these individuals [71].

2 Confidence intervals reported in the present study were computed using the MBESS package in R (see [72]).

3 It should be noted, however, that the two samples were partly overlapping, and so this study should not be considered a fully independent replication.
